# Skin renewal activity of non-thermal plasma through the activation of β-catenin in keratinocytes

**DOI:** 10.1038/s41598-017-06661-7

**Published:** 2017-07-21

**Authors:** J. H. Choi, Y. S. Song, K. Song, H. J. Lee, J. W. Hong, G. C. Kim

**Affiliations:** 1 Department of Internal Medicine, School of Korean Medicine, Pusan National University, Yangsan, South Korea; 2 Department of Oral Anatomy and Cell Biology, School of Dentistry, Pusan National University, Yangsan, South Korea; 3 Department of Biochemistry, College of Life Science and Biotechnology, Yonsei University, Seoul, South Korea; 4 Department of Electrical Engineering, Pusan National University, Busan, South Korea; 5 (Bio)medical Research Institute, Pusan National University Hospital, Yangsan, South Korea

## Abstract

For recent years, devices that generate non-thermal plasma (NTP) have been introduced into the field of dermatology. Since NTP has demonstrated strong anti-pathogenic activity with safety of use, NTP was first applied to sterilize the skin surface to aid in the healing of various kinds of skin diseases. However, the effect of NTP on skin regeneration has not yet been fully explored. In this study, the effect of NTP on the growth of keratinocytes was tested using the HaCaT human keratinocyte cell line and HRM2 hairless mice. Treatment with NTP allowed confluent keratinocytes to escape from G1 cell cycle arrest and increased the proportion of cells in S and G2 phases. In particular, NTP treatment immediately dispersed E-cadherin-mediated cell-to-cell interactions, resulting in the translocation of β-catenin to the nucleus and leading to the enhanced transcription of target genes including c-MYC and cyclin D1. Moreover, repeated treatment of the mice with NTP also stimulated epidermal expansion by activating β-catenin in the epidermal cells. The symptoms of cellular DNA damage were not detected after NTP treatment. Taken together, these results demonstrate that NTP may be employed as a new type of skin regenerating device.

## Introduction

The maintenance of healthy skin requires the continual proliferation and differentiation of the epidermal cells of the skin^1^. The turnover time of epidermal cells in adults is approximately 6–8 weeks^2^ and this renewal activity slows as the skin get older. The active growth of epidermal cells is essential for fast wound healing as well as for healthy skin tissue^3^. Therefore, skin reconstruction is important for skin care as well as skin defects resulting from injury, ulcer and tumor removal.

The proliferation of keratinocytes in the epidermis is driven by both growth factor-mediated regulation and intercellular contact-mediated regulation. In growth factor-mediated regulation, various growth factors in the dermis, such as epidermal growth factor^4^ and fibroblast growth factor 7/keratinocyte growth factor^5^, are reported to stimulate the proliferation of the cells in the stratum basal by binding to their receptors. The wnt/β-catenin signaling pathway has also been reported as one of the major regulators of the proliferation and differentiation of keratinocytes^6–8^ in hair follicles. Cellular interaction-mediated signals also play important roles in the regulation of keratinocyte growth. The interaction between integrin and extracellular molecules creates a signal that promotes the proliferation of keratinocytes in the stratum basal^9^. On the other hand, excessive cell-to-cell interactions, which usually occur in the upper layers of the stratum basal, inhibit cell proliferation, and this process is known as the contact inhibition of cell growth^10^. E-cadherin-mediated growth inhibition is well known to be involved in this process^11, 12^. E-cadherin not only plays important roles in the maintenance of homeostasis in the epidermis^13, 14^, but also has anti-proliferative functions in various cancers^15, 16^. The homophilic interaction of E-cadherins from neighboring cells stimulates the formation of an adherence junctional complex that includes α, β-catenin on its intracellular domain to form cell cytoskeleton^17^. Importantly, β-catenin is a key factor in the wnt signaling pathway and acts as a transcriptional regulator that promotes the expression of cell proliferation genes such as cyclin D1 and c-MYC^18–20^.

To date, the laser device has been regarded as the gold standard medicinal device for skin rejuvenation^21^. The strategy for the acquisition of new skin tissue involves the removal of aged skin tissue using the thermal energy of the laser, which then stimulates the remaining tissues to recover through the natural wound healing process. This method is accompanied by several adverse effects, such as pain from the heat, the risk of infection, and erythema^22, 23^. For these reasons, a new technique that can stimulate skin rejuvenation without tissue damage is needed.

Non-thermal plasma (NTP) devices have recently been introduced in dermatology as potential medicinal devices because plasma has been reported to provide various medical benefits^24, 25^. Among them, the strong antibacterial effect of NTP devices can inhibit infectious skin diseases and accelerate wound healing processes^26^. However, despite many reports, the mechanism underlying NTP-mediated regeneration of skin tissue is not fully understood. We previously reported that NTP treatment modulated skin barrier function by inhibiting E-cadherin-mediated cellular interactions^27^. Given that E-cadherin is important for the formation of the skin barrier system and the regulation of keratinocyte proliferation, it has been suggested that NTP treatment might free keratinocytes from E-cadherin-mediated growth inhibition.

In this study, we investigated the possibility that the inhibition of E-cadherin by NTP treatment could accelerate skin regeneration through the activation of β-catenin. First, the activities of E-cadherin and β-catenin in HaCaT human keratinocytes were monitored after NTP treatment. Next, the effect of NTP on the cell motility and cell cycle programs of keratinocytes under contact growth inhibition was assessed. Finally, the effect of NTP on the epidermal cell growth of normal or wounded skin was explored using HRM2 hairless mice. This study demonstrates that NTP blocks E-cadherin-mediated contact inhibition and is therefore a promising device for accelerating skin regeneration without tissue damage.

## Results

### The effect of NTP on the localization of β-catenin in cultured keratinocytes

**Figure 1:**
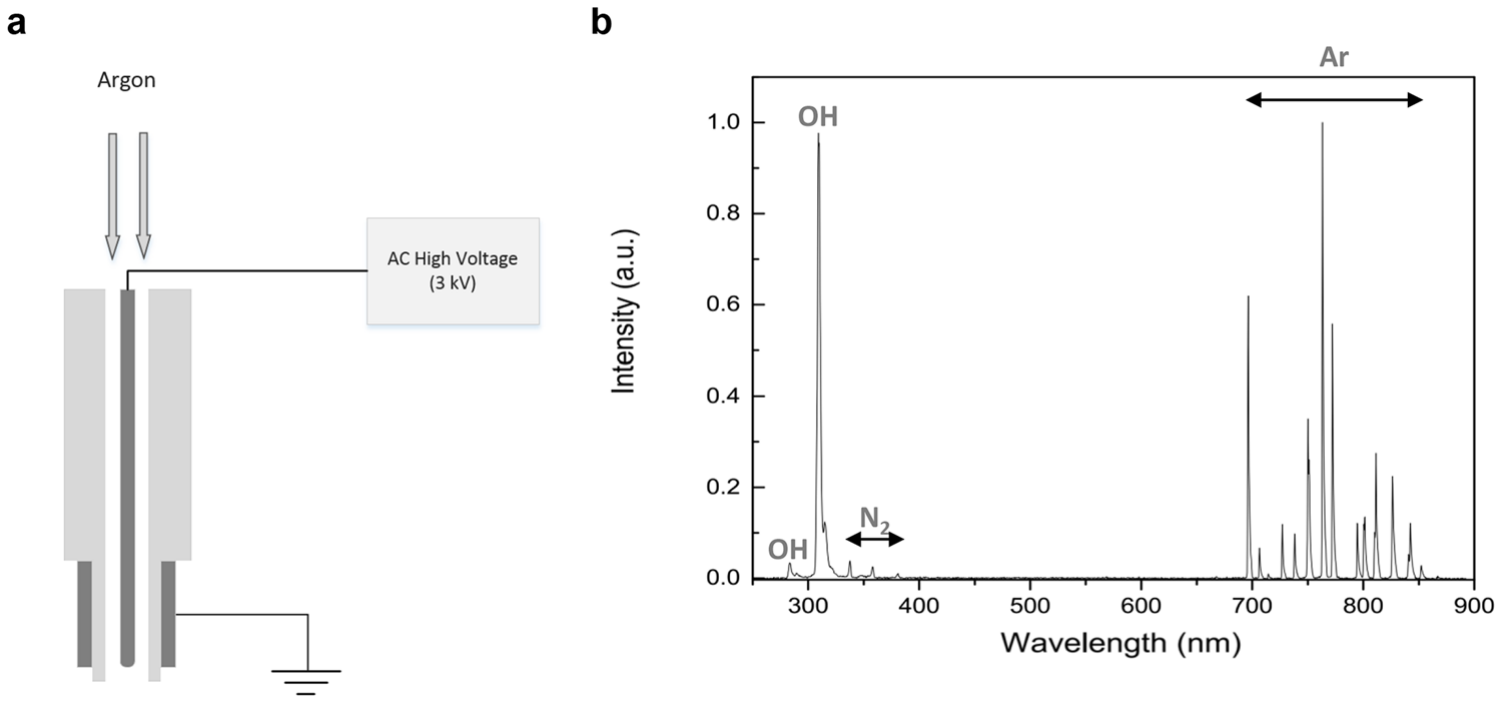
The brief introduction of NTP device. (**a**) Schematic of the plasma device which is a coaxial DBD (dielectric barrier discharge) jet type. (**b**) Optical emission spectrum of the Ar plasma from 200 to 900 nm. Ar lines, N_2_ 2^nd^ positive system and OH were observed.

**Figure 2:**
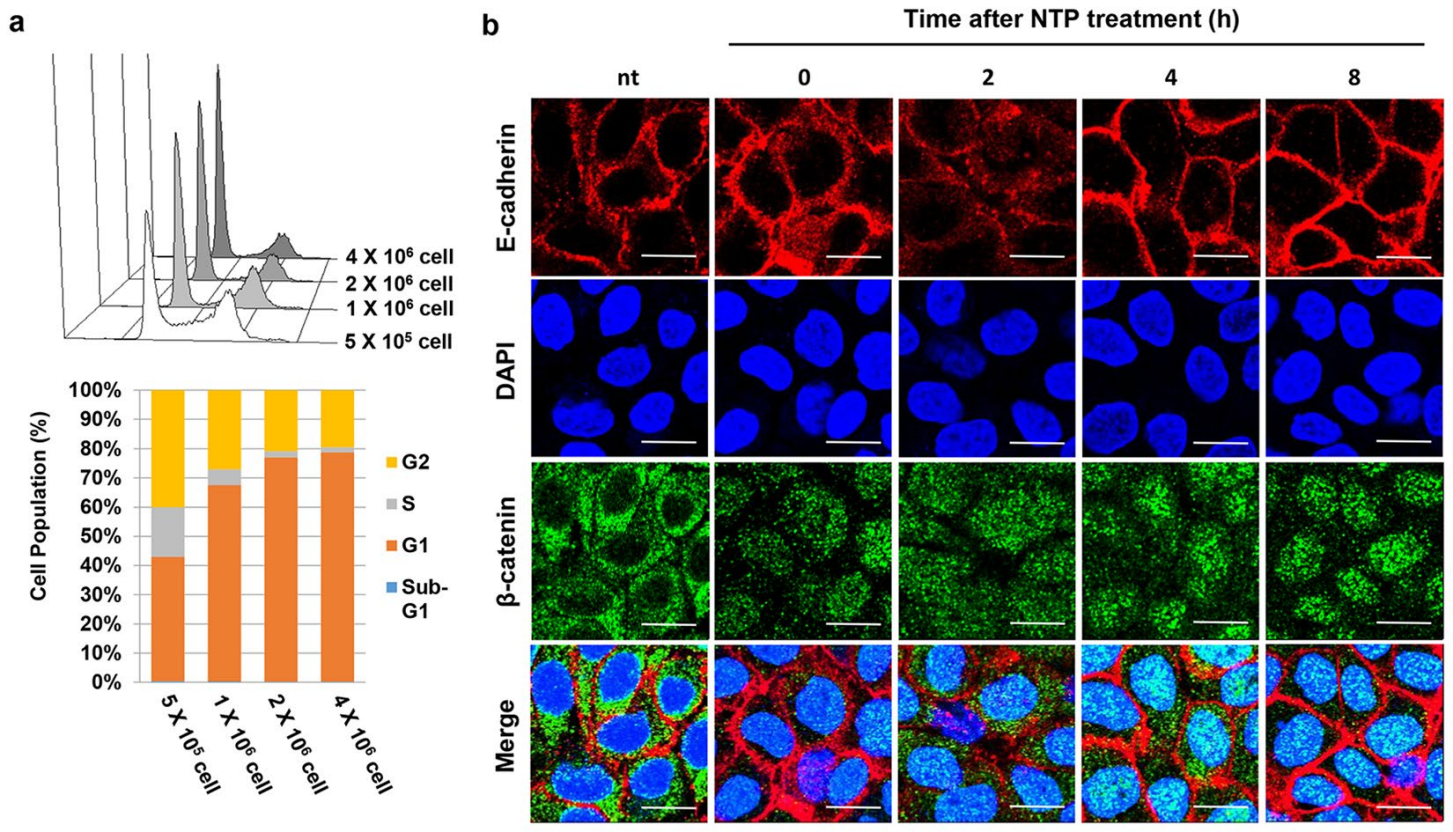
The effect of NTP on β-catenin localization in confluent HaCaT cells. (**a**) The results of FACS analysis using PI. HaCaT cells were seeded on 35 mm dish at the indicated concentration. Data shown are representative of four independent experiments. (**b**) The result of confocal imaging of immunofluorescence (Scale bar: 20 μm). Cells were treated with NTP for 5 minutes at 0, 2, 4, and 8 hours before harvesting. After fixation, cells were stained for E-cadherin (red) and β-catenin (green). DAPI was used for counter staining of the nuclei. nt: non-treated.

The DBD-type NTP generating device described earlier^28^ was slightly modified and used for this study (Fig. 1). To establish the culture conditions for HaCaT cells under contact inhibition, changes in cell cycle according to the seeded cell number were monitored (Fig. 2a). When over 2 × 10^6^ cells were seeded in 35 mm dish, the growth of HaCaT cells was inhibited and approximately 77% of the cells were arrested in G1 phase. Under these conditions, E-cadherin was well expressed around the cell boundary and β-catenin was mainly distributed in the cytosol. To test the effect of NTP on E-cadherin and β-catenin activity, the localization of these two proteins was monitored after NTP treatment (Fig. 2b). The expression of E-cadherin in cell border regions was weak at 2 hours after NTP treatment, and recovered 4 hours after NTP treatment. The translocation of β-catenin from the cytosol to the nucleus was observed after NTP treatment. The β-catenin expression was strong at 2 and 4 hours after NTP treatment, and nuclear β-catenin was weakly detected until 8 hours after the treatment.

### The effect of NTP on β-catenin-mediated gene expression

**Figure 3:**
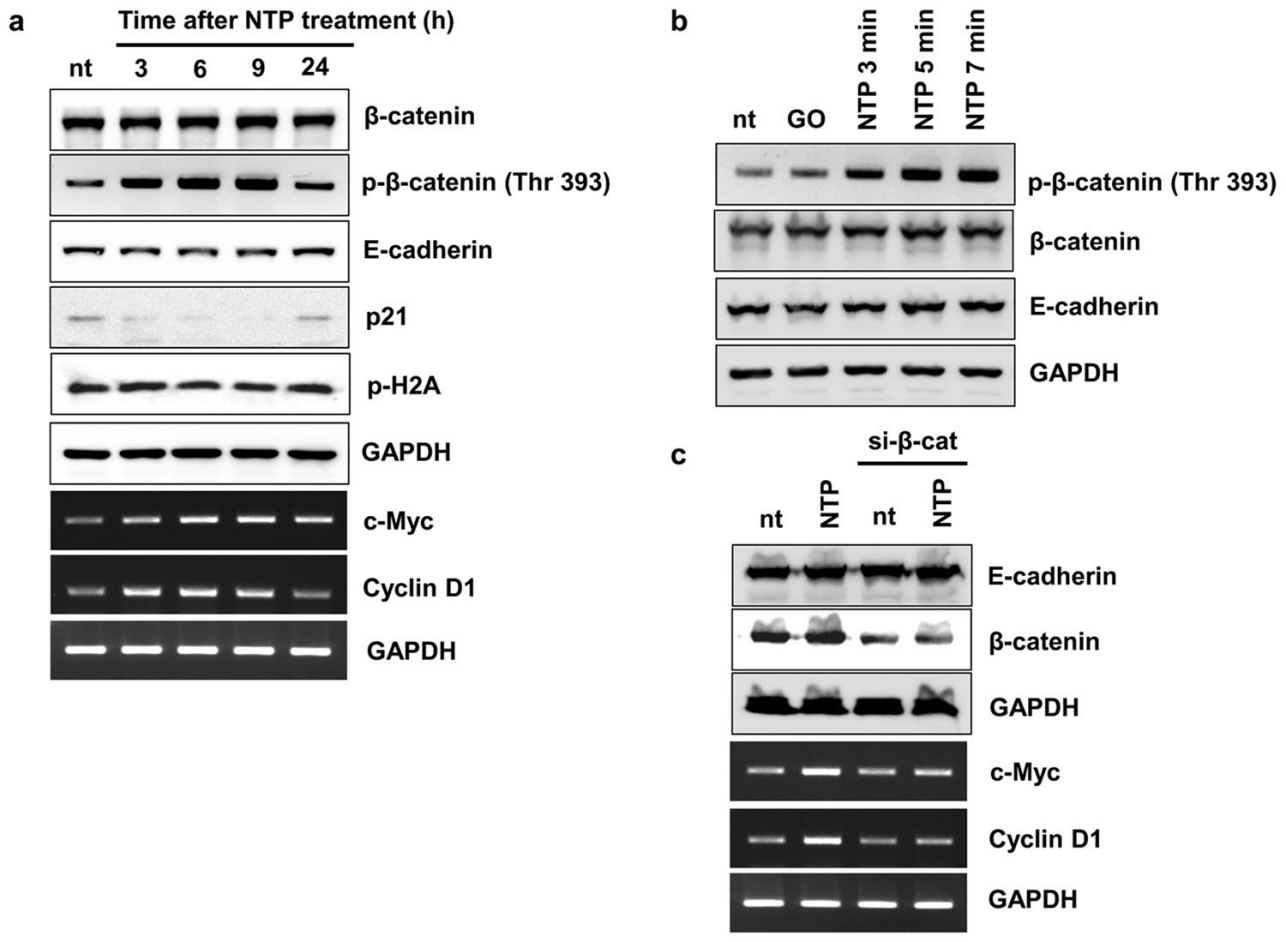
The effect of NTP on β-catenin expression in confluent HaCaT cells. (**a**) The results of Western blot and RT-PCR. Confluent HaCaT cells were treated with NTP for 5 minutes at 3, 6, 9, 24 hours before harvesting. (**b**) Confluent HaCaT cells were treated with NTP for the indicated length of time and incubated for 6 hours. After harvesting the cells, the total protein extracts were used for Western blot analysis. GO: Gas only (argon). (**c**) One day after seeding, HaCaT cells were transfected with scrambled siRNA (sc) or siRNA against β-catenin. Six hours before harvesting, cells were treated, or not, with NTP for 5 minutes. After harvesting, the total RNA and protein extracts were used for RT-PCR and Western blot analysis, respectively. Data shown are representative of three independent experiments. GAPDH was used as loading control. All images presented here are the cropped images (see Supplementary information for the original images).

To better understand the effect of NTP on HaCaT cells under contact inhibition, Western blot and RT-PCR were performed. As shown in Fig. 3a, the NTP did not modulate the total protein levels of E-cadherin and β-catenin; however, β-catenin was highly phosphorylated at Thr 393 residue after NTP treatment. The expression of p21, a key factor in cell cycle arrest, was decreased by NTP treatment; however, NTP treatment increased the expression of both c-MYC and cyclin D1, important factors in cell progression that are controlled by β-catenin. The phosphorylation of histone 2A (H2A) was not affected by NTP treatment, indicating that this did not induce DNA damage.

The phosphorylation level of β-catenin was investigated according to the length of the NTP treatment (3, 5, and 7 minutes). Six hours after NTP treatment, the expression level of phosphorylated β-catenin in HaCaT cells was increased in a time-dependent manner (Fig. 3a) while the expression levels of β-catenin and E-cadherin remained unaffected.

To test whether the increased expression levels of c-MYC and cyclin D1 were induced by the activation of β-catenin after NTP treatment, a β-catenin siRNA assay was performed. As shown in Fig. 3c, NTP treatment increased the expression of c-MYC and cyclin D1, and the transient knockdown of β-catenin partially blocked the NTP-mediated increase of c-MYC and cyclin D1 expression.

### The effects of NTP on the cell cycle and wound healing activity of HaCaT keratinocytes

**Figure 4:**
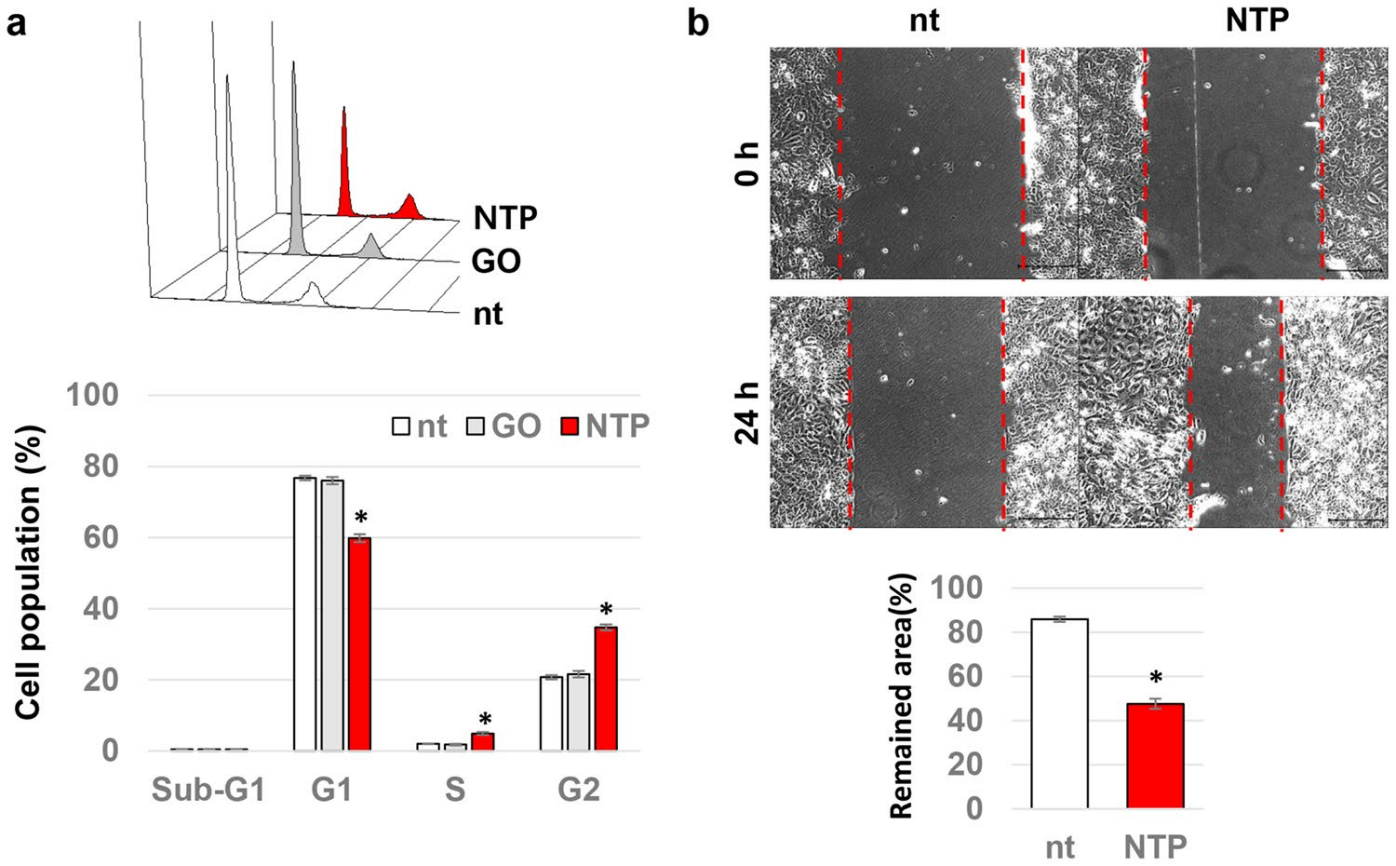
The effect of NTP on cell cycle and wound healing activities of confluent human keratinocytes. (**a**) Confluent HaCaT cells were treated with gas only (GO) or NTP for 5 minutes as indicated. After 6 hours of incubation, the cells were harvested and used for a FACS assay with PI. The percentage of cells in each of the cell cycle phases was calculated as described in the Methods section. Data shown are the averaged percentages from four independent experiments, *p < 0.05. (**b**) The results of the wound healing assay using human primary keratinocytes, HEKa. Five days after seeding, a scratch was made on each culture dish and the cells were treated, or not, with NTP for 5 minutes. Photographs of the scratch were taken at just before and 24 hours after the NTP treatment. Scale bar: 200 μm. The gap distance were calculated and presented as graph. Data shown are the averaged percentages from four independent experiments, *p < 0.001.

Next, a FACS assay using PI was performed to confirm whether NTP modulates the cell cycle of HaCaT keratinocytes. The NTP treatment reduced the proportion of G1 phase cells from 78% to 61% and simultaneously increased the cells in S and G2 phases from 1.8% to 4.9% and from 21% to 34.7%, respectively (Fig. 4a). NTP-mediated cell death was not detected. The effect of NTP on cell proliferation was also investigated through an *in vitro* wound healing assay using primary human keratinocytes. The vacant space made by the scratch was quickly filled with cells in the culture dish that was treated with NTP for 5 minutes, compared to the culture dish that was not treated with NTP (Fig. 4b).

### The effects of topical NTP treatment on β-catenin activity and the expression of cyclin A and E in mouse skin

**Figure 5:**
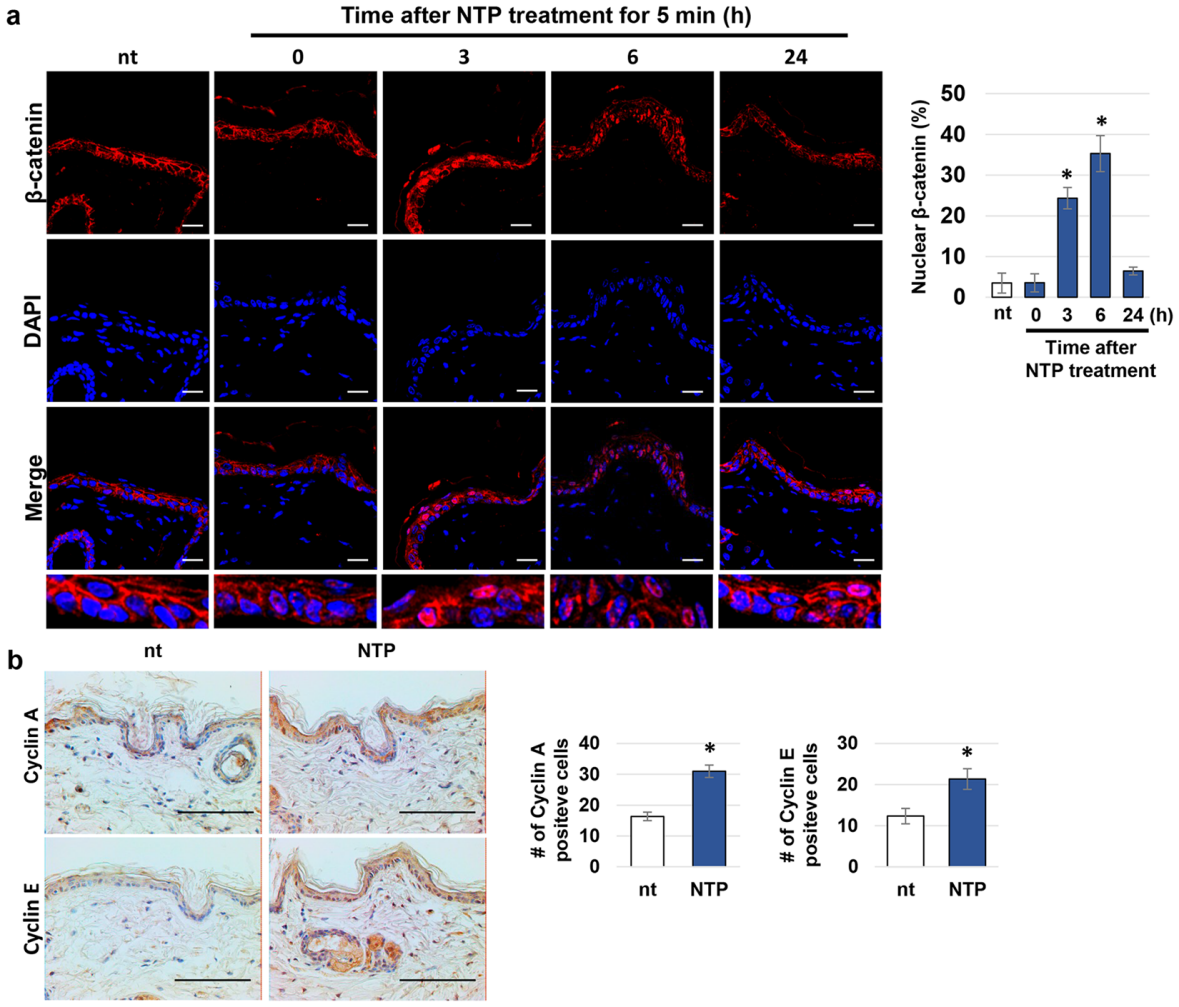
The effect of NTP on β-catenin activity in skin tissue. (**a**) Marked regions on the dorsal skin of four HRM2 mice were subjected to NTP treatment for 5 minutes at the indicated time point (0, 3, 6, and 24 hours) before sacrificing the mice. (Left panel) The skin tissue was isolated and subjected to immunofluorescence staining of β-catenin (red), DAPI counterstaining of the nuclei (blue), and confocal imaging. Scale bar: 20 μm. (Right panel) The graph showing the percent of nuclear β-catenin positive cells in epidermal tissues, *p < 0.001. (**b**) Non-treated and NTP-treated skin tissue was isolated 24 hours after treatment and subjected to immunohistochemistry against cyclin A and E. The nuclei were counterstained using hematoxylin. Scale bar: 100 μm. The number of Cyclin A and Cyclin E-positive cells were counted and represented as graphs (Right panels). Data shown are representative of three independent experiments, *p < 0.05.

To confirm whether the NTP-mediated activation of β-catenin also occurs in animal epidermis, the dorsal skin of HRM2 mice was subjected to topical treatment with NTP. The localization of β-catenin was monitored at 0, 3, 6, and 24 hours after the treatment (Fig. 5a). The expression of β-catenin was observed at the boundary of cells in non-treated mouse epidermis, whereas increased β-catenin expression in the nuclei was observed 3 and 6 hours after NTP treatment. By 24 hours after NTP treatment, the majority of β-catenin was again distributed at the boundary of cells, as for non-treated epidermis. The effect of β-catenin activation by NTP on the cell cycle in epidermis was also tested. At 24 hours after NTP treatment, nuclear cyclin A and cyclin E were both increased in the epidermis (Fig. 5b).

### The enlargement of the epidermis after repeated treatment with NTP

**Figure 6:**
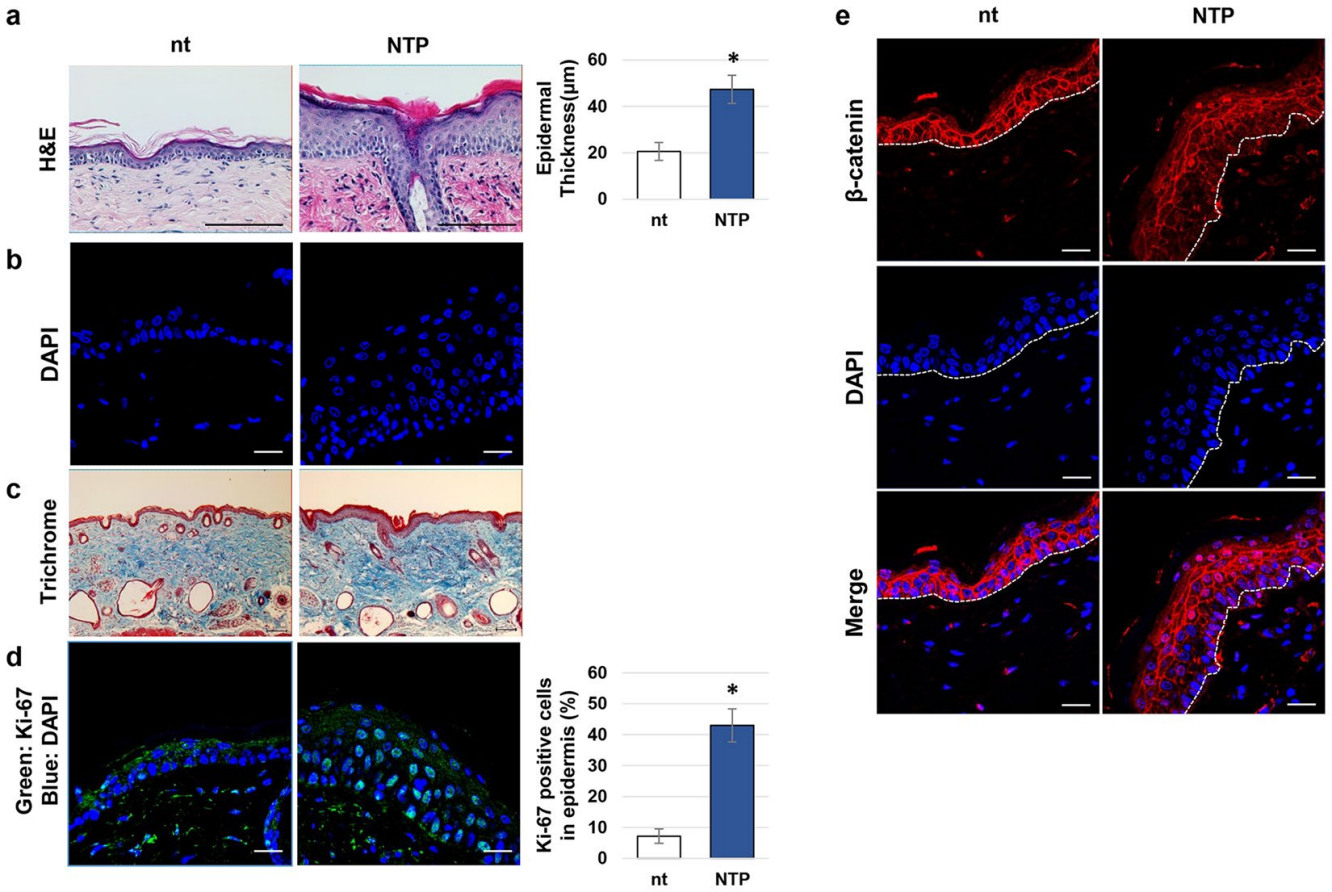
The effect of NTP on epidermal growth. HRM2 mice were subjected to NTP treatment for 5 minutes, three times per week, for 2 weeks. After sacrificing the mice, the tissues were subjected to histological analysis. (**a**) Results of H&E staining, scale bar: 100 μm. The epidermal thickness from 4 independent experiments was calculated and averaged, presented as graph (right panel), *p* < 0.01. (**b**) The results of DAPI staining, scale bar: 20 μm. (**c**) Results of trichrome staining, scale bar: 100 μm. (**d**) Results of immunofluorescence staining against Ki-67, and the percentage of Ki-67-positive cells were presented as a graph (right panel). The data shown are representative of four independent experiments. Scale bar: 20 μm, *p < 0.005. (**e**) NTP-treated or non-treated tissues (nt) were subjected to immunofluorescence staining of β-catenin (red), DAPI counterstaining of the nuclei (blue), and confocal microscopic images were taken (scale bar: 20 μm).

The dorsal skin of HRM2 mice was repeatedly treated with NTP for 5 minutes, every other day, over a period of 2 weeks. As shown in Fig. 6a, the NTP-treated epidermis layer was enlarged, becoming approximately 2.3 times thicker than the non-treated control tissue. By DAPI staining, much more nuclei were observed to form the enlarged multiple layers of epidermis (Fig. 6b). In addition, an increase in nuclear Ki-67, a marker of proliferative cells, was detected in the basal keratinocytes of NTP-treated skin tissue (Fig. 6d). Furthermore, the collagen fibers of the dermis were condensed by NTP treatment; they were stained dark pink and blue by H&E and Masson’s trichrome stain, respectively, compared with those of non-treated skin (Fig. 6c).

To investigate the relevance of NTP-mediated epidermal enlargement, the translocation of β-catenin after repeated treatments with NTP was also observed (Fig. 6e). In non-treated skin tissue, β-catenin was mainly expressed in the cell border region, even in the bottom layer of the epidermis, whereas in NTP-treated skin, β-catenin was strongly expressed in the nuclei of both the basal and upper layer cells.

### The effects of NTP treatments on cutaneous wound healing

**Figure 7:**
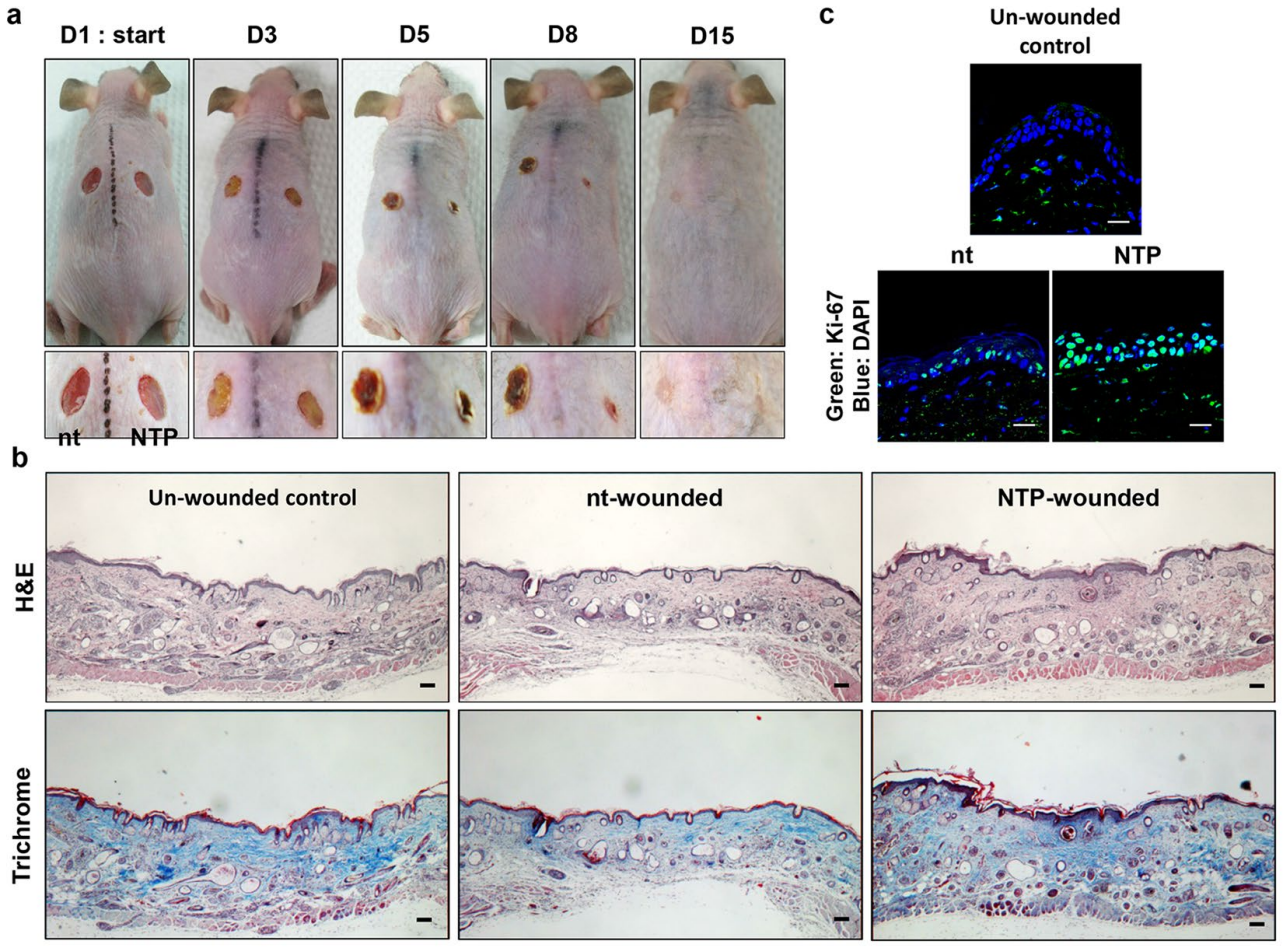
The effect of NTP on wound healing in mouse skin. (**a**) Two equal-sized wounds were created in the dorsal skin of HRM2 mice, as described in the Materials & Methods section. One of the wounds was subjected to NTP treatment for 5 minutes, three times per week, for 2 weeks. Over the course of the treatments, external changes in the wounds were monitored by taking photographs at the indicated time points. Data shown are representative of four independent experiments. (**b**) On day 15, the skin tissue of the wounds were isolated, and subjected to H&E and trichrome staining. Data shown are representative of four independent experiments, and photographs were taken at 40X magnitude. Scale bar: 100 μm. (**c**) The results of IF assay against Ki-67 using the skin tissues. Scale bar: 20 μm.

In the final part of this study, the role of NTP in re-epithelialization during the wound healing process was tested in HRM2 mice. As shown in Fig. 7a, the wounds that were repeatedly treated with NTP healed much faster than non-treated wounds. In particular, NTP treatment accelerated the shrinkage of the wound and the drop-off of the scab, so that the remaining scar size on the day 15 was smaller than that of the non-treated tissue. In tissue samples, the wound that healed naturally showed thin epidermal tissue, a low density of collagen, and a damaged muscle layer, whereas the wound that healed with NTP treatment showed an almost full recovery of epidermis, a high density of collagen, and regenerated muscle tissue (Fig. 7b). To visualize the effect of NTP on keratinocytes proliferation in wounded skin, IF assay against Ki-67 was adopted. As Fig. 7c shows, the epidermis of un-wounded skin rarely has Ki-67-positive proliferating cells, and the naturally healed skin tissue has slightly increased number of Ki-67 positive cells in epidermis than un-wounded skin. Interestingly, the epidermis of skin healed with NTP harbors greatly increased Ki-67 positive cells (about 75%).

## Discussion

This study elucidates the mechanism underlying NTP-mediated skin regeneration at both cellular and tissue levels. A plasma device for skin rejuvenation was introduced several years ago, but it was the same as ablative lasers in using high temperatures to peel off skin. As technical advances have been made in plasma science, many kinds of non-thermal plasma devices have recently been developed. Abundant studies have been reported concerning the tissue safety and medical functions of NTP, however, the effect of NTP on skin renewal are yet to be fully explored.

We previously reported that NTP treatment of keratinocytes caused the translocation of E-cadherin from cell border regions to the cytosol^27^. This suggested the possibility that keratinocytes could escape from the contact inhibition of cell growth. In the first part of this study, NTP was shown to inhibit E-cadherin-mediated cellular interactions, but these interactions were fully recovered within 4 hours (Fig. 2b). Interestingly, Haertel *et al*. also found that NTP treatment on HaCaT cell reduced the E-cadherin positive cells by FACS analysis^29^. However, they treated NTP on the cells in suspension, and the detection of E-cadherin was performed at 24 hours after the NTP treatment. Furthermore, they did not linked this phenomenon with cell proliferation. We also found that β-catenin simultaneously translocated from the cytosol to the nucleus after NTP treatment, and remained there for 8 hours. The nuclear localization of β-catenin is essential for Wnt-mediated signal transduction^20^, in which nuclear β-catenin binds to T cell factor (Tcf) and stimulates the expression of proliferative genes including cyclin D1 and c-MYC^30^. NTP treatment did not change the expression level of β-catenin but increased phosphorylation at Thr 393 (Fig. 3a and b), which is important for enhancing the transcriptional activity of β-catenin^31^. The NTP-mediated phosphorylation of β-catenin was accompanied by enhanced expression levels of both cyclin D1 and c-MYC (Fig. 3c) and decreased expression of the anti-proliferative protein p21 (Fig. 3a). Furthermore, the expression of p-H2A remained unchanged with NTP treatment, indicating that the activation of β-catenin and the induction of cell proliferation occurred without DNA damage. This finding is in a line with that of Wende *et al*., who could not find any significant increase of micronuclei after NTP treatment on HaCaT cells^32^. Generally, the cells under contact inhibition showed cell cycle arrest at G0 or G1 phase. In our results, NTP treatment allowed many of the keratinocytes arrested at G1 phase to progress through to S and G2 phases, leading to increased cell proliferation, and this was supported further by enhanced wound healing activity *in vitro* (Fig. 4).

NTP-mediated activation of β-catenin was also detected in the skin tissue of mice. Topical treatment of HRM2 mouse skin with NTP resulted in the translocation of β-catenin from cell border regions to the nucleus, and β-catenin was fully restored to its original location by 24 hours after treatment (Fig. 5a). NTP treatment of the skin clearly induced the proliferative activity of the epidermal cells. NTP treatment induced the expression of cyclins A and E in the epidermal cells and slightly increased the epidermal thickness (Fig. 5b). Since the elevated expression of cyclins A and E usually follows the induction of cyclin D during cell cycle progression from G1 to S and G2 phase^33^, these results indicate that NTP stimulates the proliferation of the epidermal cells.

NTP-mediated skin remodeling was observed with increasing treatment repeats (Fig. 6). Six lots of NTP treatment, administered over 2 weeks, were shown to increase epidermal thickness (Fig. 6a). In general, the thickness of the epidermis is maintained by balancing the proliferation and programed cell death of keratinocytes^1^, and the enlargement of the epidermis therefore indicates that the proliferative activity of the keratinocytes was increased after NTP treatment. By DAPI staining, NTP treatment was found to increase the number of nuclei in the epidermis significantly, especially in the basal layer (Fig. 6b). NTP-mediated epidermal cell growth was further confirmed by the increased expression of nuclear Ki-67 in NTP-treated tissues compared to untreated tissues (Fig. 6d). Patel *et al*. reported that nuclear Ki-67 was strongly expressed on the wound edge in mouse skin at the early stage of the natural wound healing process, and that this region then becomes a leading edge that moves towards the wound bed to cover the wound with actively proliferating keratinocytes^34^. Therefore, the increased expression of nuclear Ki-67 in the epidermis following NTP treatment indicates that this could accelerate the re-epithelialization process of wound healing. One of the most interesting findings of this study is that the NTP-mediated increase in basal cell growth occurred with the translocation of β-catenin in skin tissues. A number of papers support the possibility that nuclear β-catenin is related to epithelial cell proliferation^6, 19, 35^. In our results, since the expression pattern of β-catenin in NTP-treated tissues coincided with that of Ki-67, the activation of β-catenin by NTP could cause the epidermal proliferation (Fig. 6e). Fortunately, the repeated treatment of NTP on the mice skin did not induced cutaneous inflammation (see Supplementary Fig. S1). Furthermore, the expressions of genes related with the keratinocyte’s differentiation were unaffected by NTP-treatment (see Supplementary Figs S2 and S3). Therefore, we concluded that NTP-mediated epidermal hyperplasia does not related with psoriasis nor tumorigenesis. Mason’s trichrome staining showed that the density of dermal collagen was increased by NTP treatment (Fig. 6c). We previously observed increased collagen expression in HaCaT cells that were treated with NTP [29], which suggests that the NTP-mediated increase in the density of dermal collagen is the result of increased expression of collagen genes in keratinocytes. In the process of wound healing, increased collagen plays essential roles in the replacement of fibrin clots in the dermis, wound closure, and the movement and proliferation of epidermal cells^36^. Repeated treatment with NTP also increased the dermal fibronectin expression (data not shown). Therefore, the increased dermal collagen and fibronectin expression resulting from NTP treatment indicates that this could affect the dermal tissue recovery during wound healing.

Although the ability of NTP to promote wound healing has been reported^37, 38^, the specific mechanisms of NTP-mediated wound healing in cells and tissue are not yet fully understood. On the basis of the results obtained, we investigated the effect of NTP on wounded tissue using a mouse skin wound model. Repeated treatment of wounded tissue with NTP dramatically accelerated the wound healing process, including faster wound closure, earlier removal of the scab, and smaller scars, when compared to wounded tissue without NTP treatment (Fig. 7a). Wound healing is a complicated process consisting of three steps: inflammation, new tissue formation, and remodeling^39^. Re-epithelialization during the new tissue formation step is driven by the proliferation and migration of keratinocytes, and once the new skin barrier is formed, the scab can drop off easily. Therefore, fast removal of the scab indicates that the re-epithelialization was completed earlier due to the active cell proliferation induced by NTP. In this study, wounds healed with NTP treatments showed a thicker epidermal layer than those without NTP treatment, indicating that NTP enhanced re-epithelialization by stimulating the proliferation of keratinocytes. As Fig. 7c shows, the epidermis of naturally healed wound at 15 days after the wound formation still harbor increased number of proliferating cells than that of un-wounded skin, which means the wound healing process was not finished. Surprisingly, the epidermis healed by NTP treatment was composed of proliferating cells mostly, which demonstrate that NTP treatment accelerated re-epithelialization activity by increasing proliferation of epidermal cells. Furthermore, NTP treatment accelerated not only re-epithelialization, but also the regeneration of collagen fibers and damaged subcutaneous tissues (Fig. 7b). The increased collagen in the dermis of wounds treated with NTP coincided with the results shown in Fig. 6a. In this case, because both dermal tissue and the epidermal layer were directly exposed to NTP, fibroblasts could be affected. Given that NTP has been reported to increased collagen synthesis from fibroblasts^40^, the NTP-mediated increase in collagen in the dermis could result from the activation of fibroblasts as well as keratinocytes.

To date, many methods have been proposed to target the proliferation of keratinocytes but there have been no skin renewal techniques that regulate cell contact inhibition. Here, NTP treatment was shown to not only stimulate epidermal cell growth by down-regulating E-cadherin and stimulating β-catenin without skin damage, but also to accelerate the dermal remodeling of wounds. NTP treatment can therefore be helpful for accelerated wound healing and the maintenance of healthy skin. Furthermore, the molecular mechanism elucidated in this study for the effect of NTP on cutaneous wound healing might also be applicable for the wound healing of other organs.

## Methods

### Cell culture

HaCaT human keratinocytes were cultured in Dulbecco’s modified Eagle’s medium (DMEM, Gibco^®^) supplemented with 10% (V/V) fetal bovine serum (FBS, HyClone^®^) and 1% antibiotics (Gibco^®^). Cells were sub-cultured on every other day to maintain a single layer of cells. For *in vitro* experiments, cells were seeded at a concentration of 2 × 10^6^ cells for a 35 mm dish and the treatments were undertaken 2 days later, unless otherwise described.

### Non-thermal plasma device

We used a type of low-frequency argon plasma device as described earlier^28^. A schematic diagram of the device is given in Fig. 1a. The plasma source is composed of ceramic tube as a dielectric and two electrodes, which is a conventional dielectric barrier discharge (DBD) type. The outer electrode is grounded and a sinusoidal high voltage is applied to the inner titanium electrode. Argon gas flows at 3 slm (standard liter per minute) between inner electrode and ceramic tube. The argon plasma jet is generated using a 15 kHz sinusoidal high voltage of 3 kVpp. The discharge power of the argon plasma jet is approximately 1 W. For analyzing the properties of the plasma from the device, optical emission spectroscopy (OES) was observed by the OES device (USB2000+, Ocean Optics, USA) in the range from 200 to 900 nm (Fig. 1b). The optical emission spectrums were detected 20 mm away from the exit of the plasma nozzle and with 0.5 ms exposure time. As a result shows, hydroxyl radicals and argon ions were generated effectively.

### Reverse transcription-polymerase chain reaction (RT-PCR)

Total RNA was extracted from the cells and 1 μg of RNA was then subjected to RT-PCR as described previously^40^. The primer sequences used for this study included: c-MYC AAGAGGTGCCACGTCTCCAC and GTCACGCAGGGCAAAAAAG for c-MYC; GCCCTCTGTGCCACAGATG and GCGCAGGCTTGACTCCAG for cyclin D1; and ACTGGCATGGCCTTCCGT and CCACCCTGTTGCTGTAGCC for *GAPDH*.

### Western blot analysis

In brief, cells were harvested and 30 μg of the total protein extracted from each sample was subjected to SDS-PAGE and Western blot. The antibodies used for this study were E-cadherin, β-catenin, phosphorylated β-catenin (Thr393), p21, GAPDH (Santa-Cruz Biotechnology), and phosphorylated H2A (Cell Signaling). A detailed description of the SDS-PAGE and Western blot procedures used in this study can be found elsewhere^40^.

### Fluorescence associated cell sorter (FACS) analysis

The proportion of cells in sub-G1, G1, S, and G2 phases of the cell cycle was quantified by flow cytometry. In brief, cells were fixed with ethanol and their nuclei stained by propidium iodide. The stained cells were sorted using a FACscan flow cytometer (Becton Dickson Co., CA, USA) and DNA histograms were analyzed using WinMDI software.

### Wound healing assay in cell culture

For testing the wound healing activity *in vitro*, HEKa human primary keratinocytes were purchased from Thermo Fisher Scientific (MD, USA) and used for this experiment. Five days after seeding, scratches of equal size were created in confluent HEKa cells using a 1 ml pipette tip. The cells were then treated, or not, with NTP for 5 minutes and initial photographs were taken of the artificial wounds using a light microscope equipped with a digital camera. The cells were then washed, given fresh growth media, and incubated for a further 24 hours in the cell culture chamber. After incubation, photographs were again taken and the recovery of the wounds assessed by comparing the initial and end point photographs.

### Mouse control

To test the effect of NTP on skin tissue, 5-week-old male HRM2 melanin-possessing hairless mice were obtained from Central Laboratory Animal Inc. (Seoul, Korea), housed in a controlled room (25 ± 1 °C, 53 ± 5% humidity), and fed a standard laboratory diet. All experimental protocols regarding animal care were performed according to the guidelines and regulations of the Animal Ethics Committee, Pusan National University (PNU-2017-1446).

### The effect of NTP on β-catenin in skin tissue

Mice (n = 4) were anesthetized and 4 regions of dorsal skin were marked on each. These regions were subjected to 5 minutes of NTP treatment at 0, 3 hours, 6 hours, or 24 hours prior to the mice being sacrificed. Skin tissues were then isolated using a 6-mm biopsy punch and subjected to histological analysis.

### The effect of NTP on skin tissue remodeling

Mice (n = 4) were subjected to repeated treatments with NTP. After anesthetization (intraperitoneal injection of a ketamine/xylazine cocktail), two regions of dorsal skin on each mouse were marked bisymmetrically. One of these regions was subjected to 5 minutes of NTP treatment three times a week, for 2 weeks, while the other region was used as a non-treated control. One day after the final treatment, the mice were sacrificed and the skin tissues were subjected to the histological tests.

### Cutaneous wound healing

The mice (n = 4) were anesthetized, and two same-sized bisymmetric wounds were created using a 6-mm biopsy punch. One of these wounds was subjected to a total of seven NTP treatments, with one being administered every other day. Photographs of the wounds were taken immediately before each of these treatments. On day 15, the mice were sacrificed and the healed skin tissues were isolated. To prevent infection of the wounds, the mice were individually housed in microisolator cages in an isolated animal care facility devoid of known mouse pathogens, and maintained in a fresh condition.

### Histological assays

The skin samples were fixed in 4% paraformaldehyde for 24 hours, and then embedded in paraffin. Sections of the skin (5 μm) were stained with hematoxylin and eosin (H&E) or Masson’s trichrome to monitor the histological changes in the skin.

### Immunohistochemistry

Immunostaining was performed using antibodies against cyclin A, cyclin E (Santa Cruz Biotechnology), with an ABC alkaline phosphatase staining system (Vector Laboratories) and 3,3′ diaminobenzidine (DAB) as the staining substrate. The nuclei of skin tissue were counterstained using hematoxylin.

### Immunofluorescence

Staining of β-catenin within the skin tissue was performed using a rabbit polyclonal anti– β-catenin antibody (Santa Cruz Biotechnology), Ki-67 (Abcam Inc., Cambridge, UK) and an Alexa Fluor 594-conjugated goat anti-rabbit antibody (Invitrogen). The nuclei within the tissues were counterstained using hematoxylin or 4′,6-diamidino-2-phenylindole (DAPI).

### Data analysis

Data are presented as the mean ± SD of at least 3 independent experiments. Two-tailed Student’s *t* tests were used to assess statistical significance for differences between means, and the threshold for significance was set at *p* < 0.05.

### Study approval

All experimental protocols for animal care were reviewed and approved by Animal Ethics Committee of Pusan National University (PNU-2017-1446) and all animal procedures were performed in accordance with the relevant guidelines.

## Electronic supplementary material

Supplementary Information
