# Supplementary material for: Skin renewal activity of non-thermal plasma through the activation of β-catenin in keratinocytes
Source: Sci Rep. 2017 Jul 21;7:6146. doi: 10.1038/s41598-017-06661-7 (PMC5522407; doi:10.1038/s41598-017-06661-7)

Title:
Skin renewal activity of non-thermal plasma through the activation of β-catenin in keratinocytes

Authors: J.H. Choi[1,2], Y.S. Song[1], K. Song[3], H.J. Lee[4], J.W. Hong [1,5*]and G.C. Kim[2*]

Affiliations:
[1] Department of Internal Medicine, School of Korean Medicine, Pusan National University
[2] Department of Oral Anatomy and Cell Biology, School of Dentistry, Pusan National University
[3] Department of Biochemistry, College of Life Science and Biotechnology, Yonsei University
[4] Department of Electrical Engineering, Pusan National University
[5] (Bio)medical Research Institute, Pusan National University Hospital

***Corresponding authors:**

Jin-Woo Hong, Department of Internal Medicine, School of Korean Medicine, Yangsan Campus of Pusan National University, Beomeo-ri, Mulgeum-eup, Yangsan-si, Gyeongsangnam 626-870, South Korea, e-mail: jwhong@pusan.ac.kr

Gyoo-Cheon Kim, Department of Anatomy and Cell Biology, School of Dentistry, Yangsan Campus of Pusan National University, Beomeo-ri, Mulgeum-eup, Yangsan-si, Gyeongsangnam 626-870, South Korea, e-mail: ki91000m@pusan.ac.kr

Supplementary Figure S1

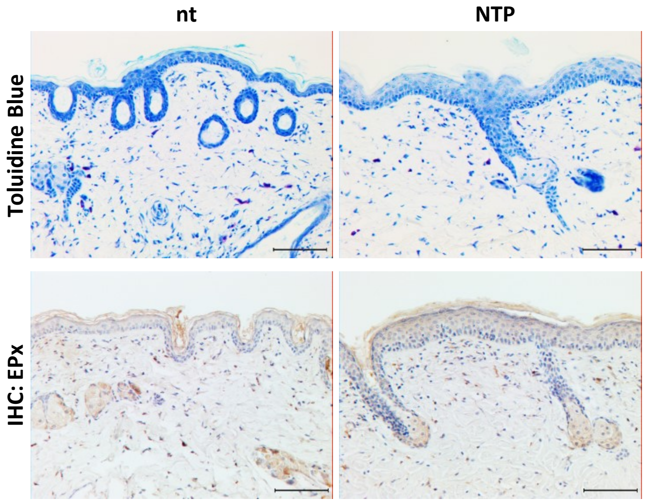

Supplementary Figure S1: The effect of NTP treatment on cutaneous inflammation: Dorsal skin of HRM2 mice were subjected to NTP treatment for 5 minutes, three times per week, for 2 weeks. After sacrificing the mice, the tissues were subjected to specific staining against mast cells (Toluidine Blue) and eosinophils (IHC:Epx) respectively. Scale bar: 100 µm.

Supplementary Figure S2

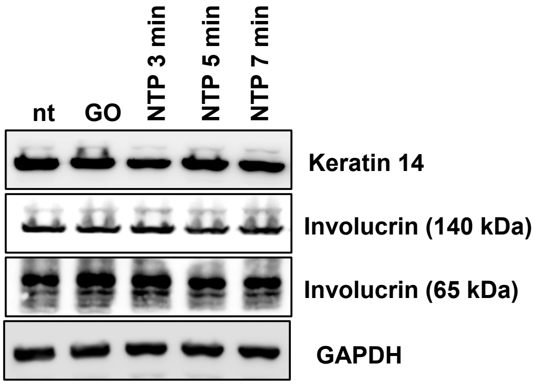

Supplementary Figure S2: The effect of NTP treatment on the expressions of genes related with keratinocyte's differentiation. Confluent HaCaT cells were treated with NTP for the indicated length of time and incubated for 6 hours. After harvesting the cells, the total protein extracts were used for Western blot analysis. **GO**: Gas only (argon).

Supplementary Figure S3

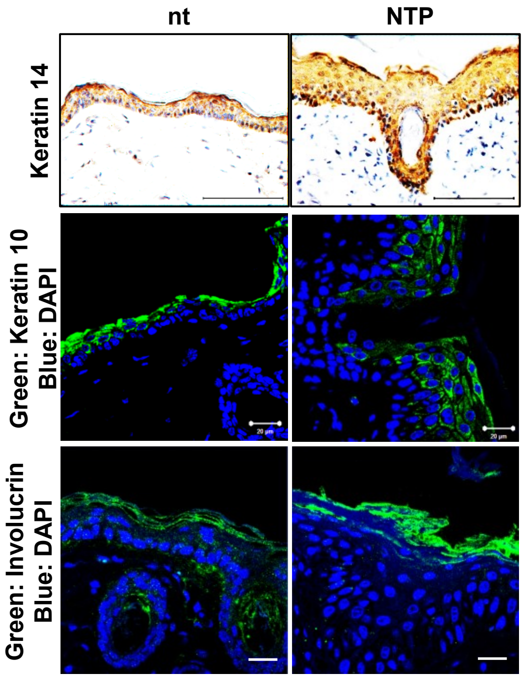

Supplementary Figure S3: The effect of NTP treatment on the differentiation of keratinocytes within HRM2 mice skin. Dorsal skin of HRM2 mice were subjected to NTP treatment for 5 minutes, three times per week, for 2 weeks. After sacrificing the mice, the tissues were subjected to IHC against Keratin 14 (scale bar: 100 μm) or IF against Keratin 10 and involucrin (scale bar: 20 μm).

Un-cropped Western blot images of Figure 3a

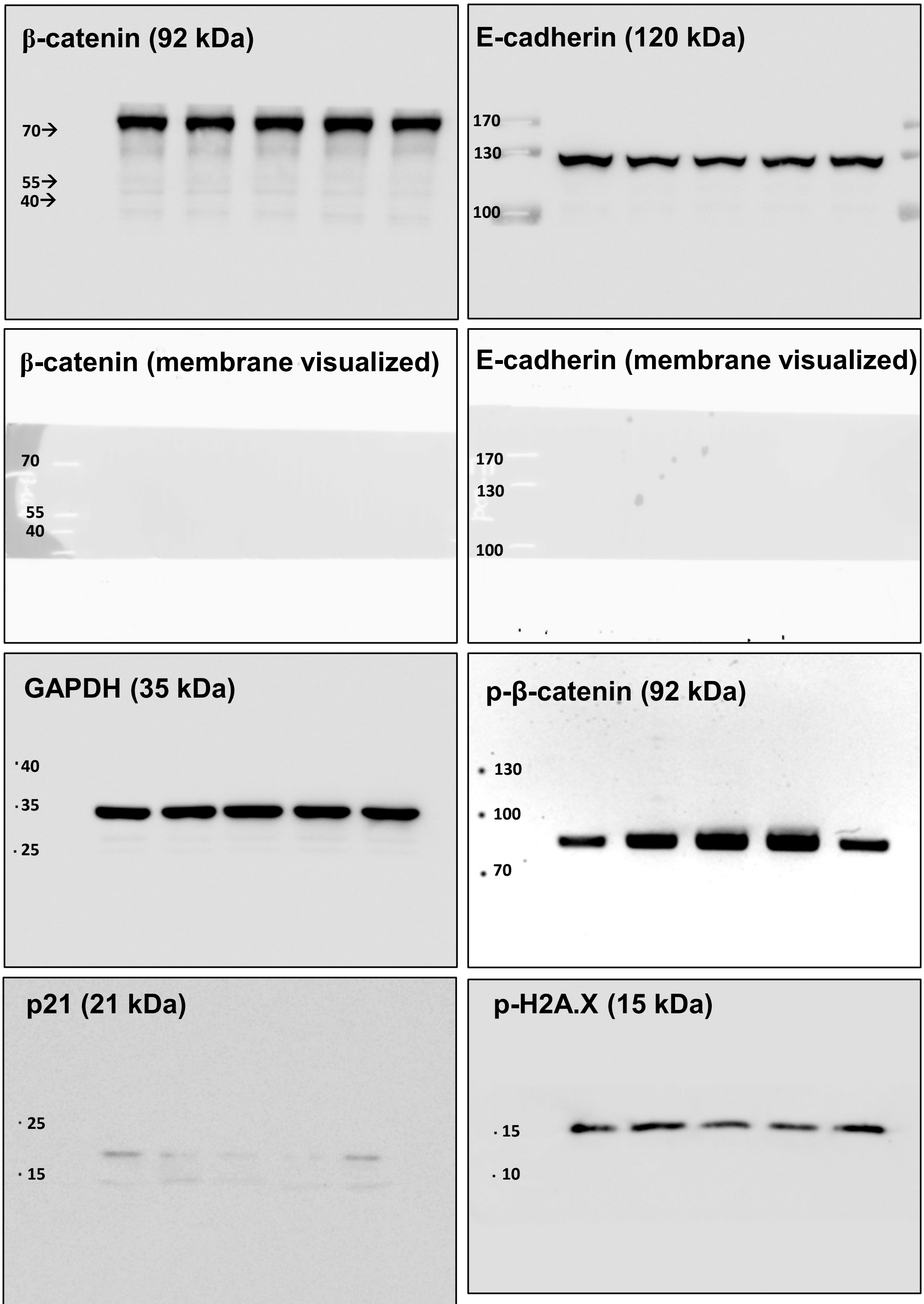

Un-cropped Western blot images of Figure 3b

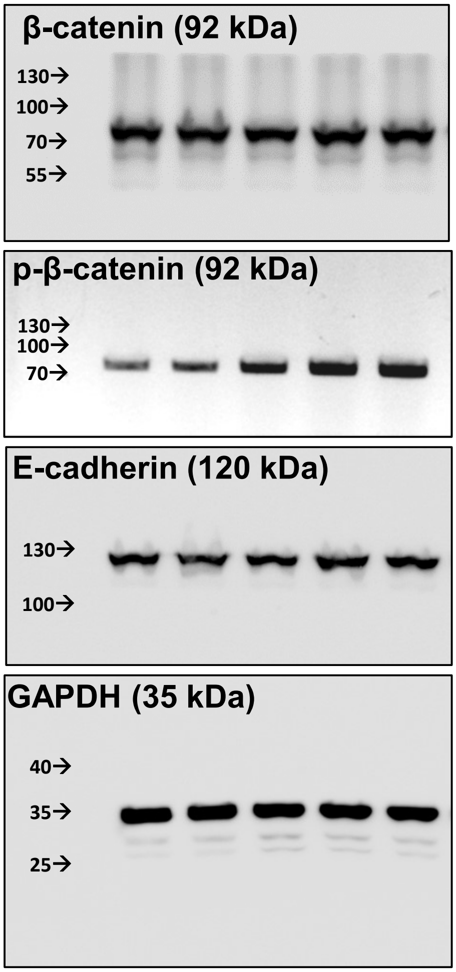

Un-cropped Western blot images of Figure 3c

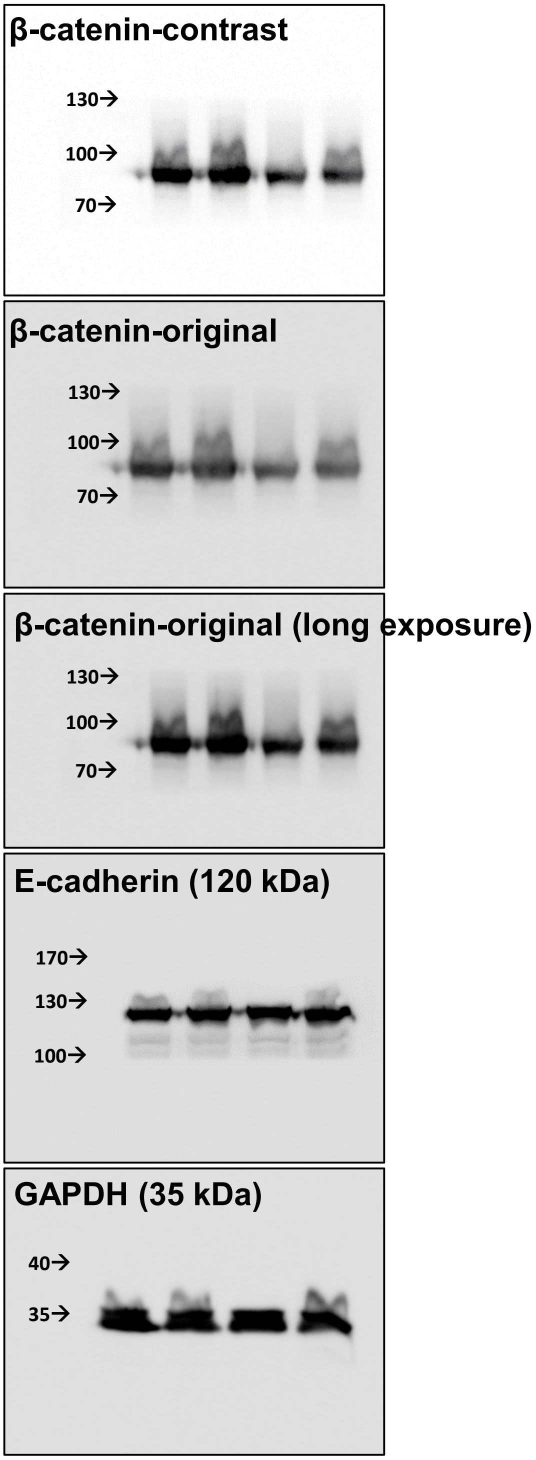

Full-gel images of Figure 3a

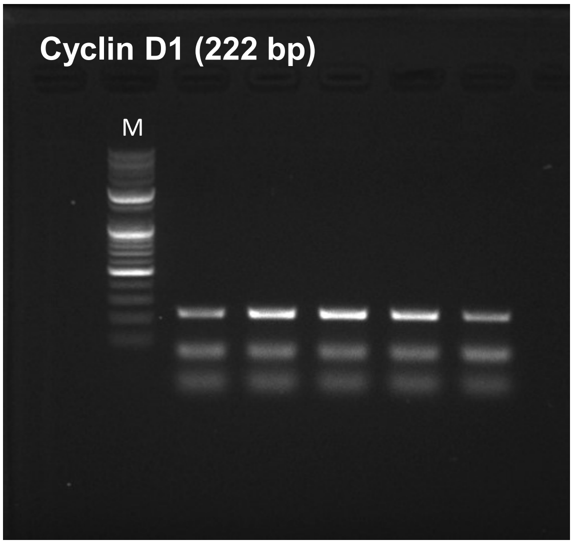

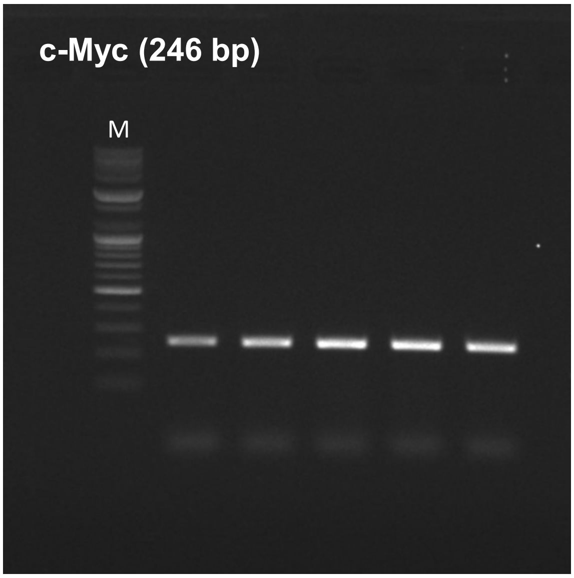

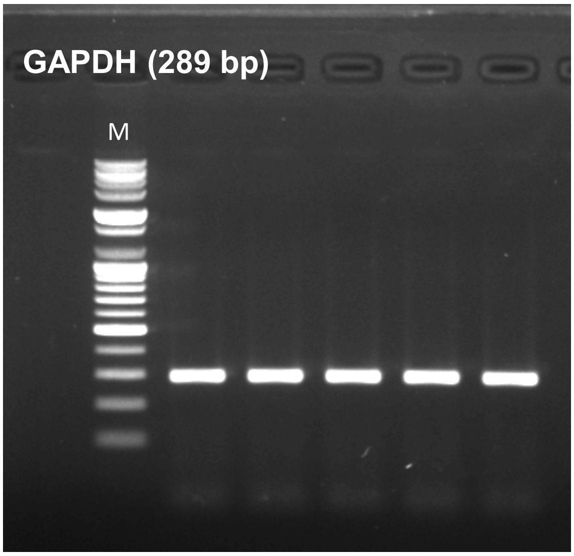

Full-gel images of Figure 3c

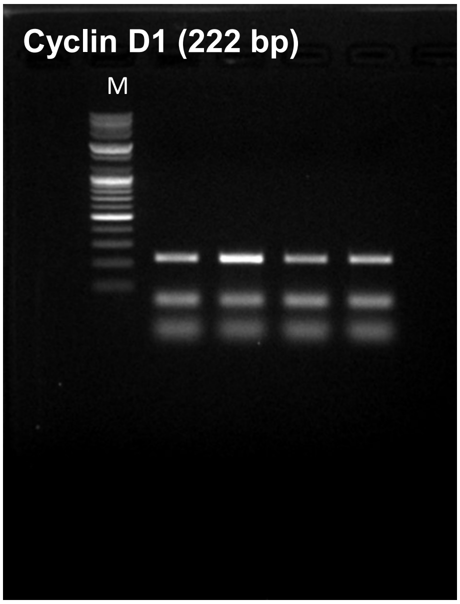

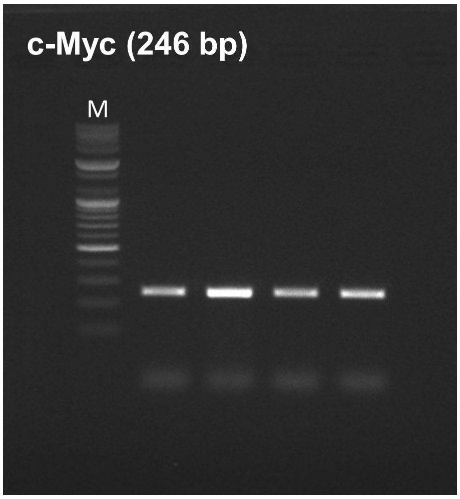

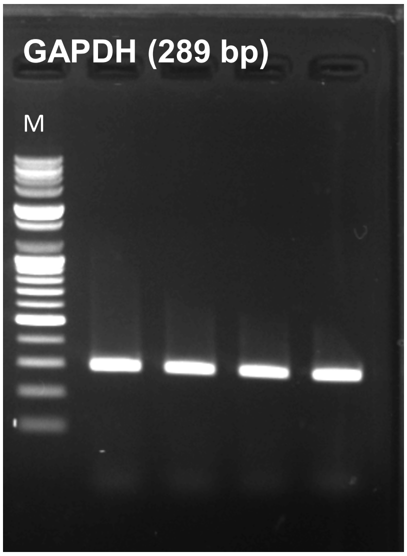

Supplement: Supplementary file 1 — Supplementary Information [file 41598_2017_6661_MOESM1_ESM.pdf]
